# Research progress of neuron-specific enolase in cognitive disorder: a mini review

**DOI:** 10.3389/fnhum.2024.1392519

**Published:** 2024-07-08

**Authors:** Fang Liu, Haiyan Li, Xiaqing Hong, Ying Liu, Ze Yu

**Affiliations:** ^1^Department of Integrated Chinese and Western Medicine/Geriatrics, Zhoushan Hospital, Wenzhou Medical University, Zhoushan, Zhejiang, China; ^2^Department of Clinical Medicine, Zhoushan Hospital, Wenzhou Medical University, Zhoushan, Zhejiang, China; ^3^Laboratory of Cytobiology and Molecular Biology, Zhoushan Hospital, Wenzhou Medical University, Zhoushan, Zhejiang, China

**Keywords:** neuron-specific enolase, glycolysis, cognitive disorder, Alzheimer's disease, nerve cells

## Abstract

Numerous studies have demonstrated that neuron-specific enolase (NSE) serves as a distinctive indicator of neuronal injury, with its concentration in blood reflecting the extent and magnitude of nervous system damage, and the expression of serum NSE is correlated with cognitive dysfunction. The assessment of NSE holds significant importance in diagnosing cognitive dysfunction, assessing disease severity, predicting prognosis, and guiding treatment. In this review, the research progress of NSE in cognitive dysfunction was reviewed, and the value of serum NSE level in predicting disease severity and prognosis of patients with cognitive dysfunction was discussed.

## 1 Introduction

### 1.1 Overview of NSE

In 1965, scientist Moore made the initial discovery of a soluble protein with acidic properties that is extensively present in neural tissues of the brain, but has limited presence in non-neural tissues. This protein, known as 14-3-2 protein or NSE, is a macromolecular substance with minimal presence in normal peripheral body fluids (Bock and Dissing, 2010). NSE exhibits the highest distribution in brain tissue, constituting ~1.5%−3.0% of all soluble proteins in brain nerve tissue and accounting for 40%−65% of enolase in the human brain cortex. The gray matter of the brain harbors a substantial population of neurons, resulting in a heightened concentration of NSE. In contrast, the peripheral nerves exhibit a mere 1%−10% of the NSE levels observed in the central nervous system. Consequently, gray matter exhibits the highest NSE content (Hein-Née et al., 2008). The amount of NSE in the blood is at least 30 times lower than in the brain. When brain tissue is damaged by ischemia, poisoning or trauma, the integrity of the cell membrane is destroyed and NSE is released. The release of NSE into the cerebrospinal fluid and subsequent entry into the bloodstream, resulting from the breakdown of the blood-brain barrier, serves as a foundation for monitoring alterations in blood NSE levels following brain tissue injury, as evidenced by findings from fundamental research studies (Angelov et al., 1994). NSE serves as a distinctive indicator of nerve injury and assumes a crucial role in the regulation of nerve cell growth and development, owing to its significant nerve specificity as an influential enolase in the glycolysis process (Hafner et al., 2012). Once a neuron is damaged, it will rapidly increase the rate of NSE synthesis by nerve cells and play a compensatory role in protecting and repairing damaged nerve. Under the action of pyruvate kinase, NSE forms ATP and improves the hypoxia state of the nerve cell source (Díaz-Ramos et al., 2012).

For eukaryotic cells, there are three enolase subtypes encoded by different genes and expressed tissue-specific; Alpha enolase (ENO1) is universally expressed, gamma enolase (ENO2) is only found in neurons, and beta enolase (ENO3) is only found in muscles. Enolase exists in dimer form, and its function depends on the natural cofactor Mg+ to regulate the conformation and catalytic activity of the enzyme (Isgrò et al., 2015). In the brain, NSE is expressed as gamma-γ on neurons and alpha-γ on microglia, astrocytes, and oligodendrocytes. NSE levels are thought to be low in the embryonic brain and may increase as neurons mature morphologically and functionally (Schmechel et al., 1980). Non-neural enolase (NNE, α-α dimer) is observed on neural tissue in the early stages of development, but is gradually transformed into gamma-γ and α-γ isoforms (NSE) as neuronal and glial cells differentiate and mature (Tanaka et al., 1985). This localization in neurons and glial cells suggests that NSE may exert inflammatory and neurotrophic activities to regulate neuronal growth, differentiation, survival, and death. NSE is thought to have different roles, including roles in glycolysis and gluconogenesis pathways, nerve cell differentiation, activation, and proliferation through PI3K/Akt and MAPK/ERK signaling pathways (Hafner et al., 2012). In addition, NSE plays a role in the activation of the RhoA kinase pathway, which can lead to neurodegeneration or neuroprotection depending on the strength of the signal (McCoy et al., 2023).

**Figure 1:**
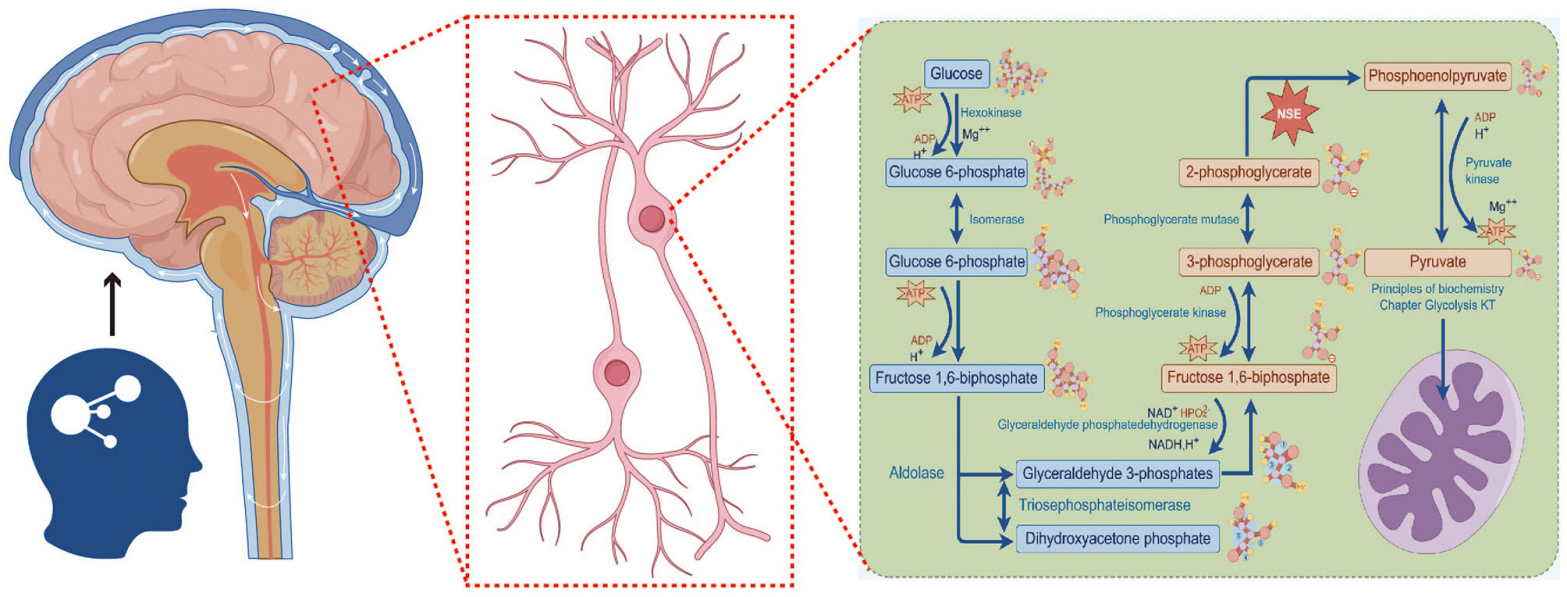
Function of NSE in glycolysis of nerve cells.

As shown in Figure 1, NSE is a vital glycolytic enzyme, and its physiological function is to regulate the growth and development of nerve cells and participate in energy metabolism of nerve cells. NSE is positively correlated with hyperphosphorylated Tau protein, so the more severe the cognitive impairment, the higher serum level of NSE (Schmidt et al., 2014).

### 1.2 Cognitive dysfunction, dementia and autism spectrum disorder

Cognitive function is an important part of the higher neural activities of the cerebral cortex, which consists of memory, computation, time and space orientation, structural ability, executive ability, language understanding and expression. Cognitive impairment is the impairment of cognitive function to various degrees due to various reasons, including mild cognitive impairment (MCI) to dementia (Morley, 2018).

Individuals with MCI typically experience subjective cognitive complaints and impairment in one or more cognitive domains, while still maintaining overall cognitive function and performing daily activities normally. The main subtypes of MCI are simple amnestic MCI (a-MCI), multiple cognitive domains with varying degrees of impairment (md-MCI), and single non-memory cognitive impairment (sd-MCI) (Petersen, 2004). Dementia is a serious and persistent cognitive disorder, dementia is caused by brain lesions, in addition to cognitive decline, but also accompanied by delusions, personality changes and other symptoms (Juva et al., 1994). Alzheimer's disease (AD) and vascular dementia (VaD) are two common types of senile dementia. Among them, AD is the main degenerative dementia, accounting for 50%−70% of all types of dementia, and VaD is the most common non-degenerative dementia, accounting for 15%−20% of all dementia patients.

Autism spectrum disorder (ASD) is a neurological developmental disorder occurring in early childhood, characterized by social communication disorders, speech and non-verbal development disorders, narrow range of interest, and repetitive and stereotypical behaviors (Sacrey et al., 2014). Most children are accompanied by varying degrees of intellectual retardation. These symptoms are often life-long. The cognitive impairment of ASD is manifested in intellectual impairment, memory impairment (selective deficiency in episodic memory), language communication impairment, face recognition, emotional cognition and social cognition impairment, temporal cognition impairment, and executive function impairment.

## 2 Expression of NSE in cognitive dysfunction

Butterfield et al. speculated that elevated enolase levels found in the brains of MCI, early-onset AD, and AD could be attributed to excitotoxicity, hypoxia, and/or oxidative stress-induced neuronal and/or glial intracellular survival pathways. Extracellular tissue PGn activator (tPA) cleavage with membrane-resident enolase-bound plasminogen (PGn) stimulates fibrinolytic activation of mitogen-activated protein kinase (MAPK), extracellular signal-regulated kinase 1/2 (ERK1/2) pro-survival pathways, and thereby upregulates transcription of glycolytic enzymes (such as enolase), to counteract low metabolic imbalances in adenosine triphosphate (ATP) and critical ion gradients, and possibly save cells from apoptotic death. Multiple studies have shown that NSE is highly expressed in cognitive dysfunction. Regulated expression of NSE may promote neuronal survival through cell survival pathways (neuroprotection or regeneration) (Butterfield and Lange, 2009).

### 2.1 Expression of NSE in MCI

MCI is characterized through cognitive deficits while maintaining independence in daily activities, and is recognized as an initial phase in various neurodegenerative dementia syndromes. Maryna et al. assessed serum NSE levels in 158 individuals with MCI and 82 healthy controls. The results showed that the serum NSE level was higher than that of the control group, suggesting that the increase of serum NSE level may indicate the neuronal damage in the patients with MCI (Polyakova et al., 2022). Meanwhile, Tian et al. studied the effect of cognitive function training intervention on elderly patients with MCI, and compared the serum NSE levels assessed by the brief mental state Examination (MMSE), the Montreal Cognitive Assessment (MoCA) and the two time points before and 6 weeks after the intervention. Results After intervention, MMSE score and MoCA score of both groups were significantly improved, and the score of intervention group was significantly higher than that of intervention group. The level of serum NSE in the intervention group was significantly decreased. This research discussed the reduction of brain tissue damage in elderly patients with MCI through the implementation of cognitive training programs by detecting changes in serum NSE (Tian et al., 2022).

### 2.2 Expression of NSE in vascular cognitive dysfunction

VaD is a syndrome of severe cognitive impairment caused by cerebrovascular disease. At present, the mainstream view is that cerebral hypoperfusion is the core cause of VaD (Rajeev et al., 2022). White matter injury and cerebrovascular diseases and the resulting ischemia or bleeding are also key causes of VaD (Hu et al., 2021).

Among the imaging markers of cerebral small vessel disease are white matter hyperintensities (WMHs). Stroke and CSVD impair neurovascular function, leading to BBB dysfunction, neurovascular discoupling, hypoperfusion, inflammation, and neuron loss. Ischemic and hemorrhagic lesions are caused by these pathological events, and cognitive impairment is closely related to them (Lecordier et al., 2021). Data obtained by Wallin et al. (1999) as early as the late 20th century led them to conclude that NSE in CSF is a marker of chronic neuronal degeneration in VaD. Subsequent research showed a correlation between increased serum levels of NSE in patients and the severity of arterial hypertension (AH), a prominent risk factor for cerebral small vessel disease (CSVD) and white matter hyperintensities (WMHs) (González-Quevedo et al., 2011). Dobrynina et al. (2023) did not find evidence to suggest that alterations in blood NSE levels were unique to cerebral small vessel disease (CSVD) or its closely related condition, arteriolosclerosis (AH), a finding that contradicts some prior research. Conversely, the research identified that an elevated blood/cerebrospinal fluid NSE ratio is a valuable tool in distinguishing VaD from AD.

Shen and Gao (2015) observed the relationship between cognitive impairment and serum NSE after stroke. A total of 42 patients with dementia after stroke were selected as the VaD group and 38 patients without dementia after stroke as the control group. Serum NSE content in the VaD group at 3 days, 3 months, and 6 months after stroke was significantly higher than that in the control group (*p* < 0.01), and had an increasing trend (*p* < 0.05). These results indicate that NSE can effectively reflect the degree of nerve cell injury and nerve cell death in patients with cognitive impairment after stroke, and can be used as a biochemical index to judge the cognitive dysfunction of patients.

Changqin Li selected 150 patients with vascular cognitive impairment and divided the 150 patients into MCI group (69 cases), moderate cognitive impairment group (45 cases) and severe cognitive impairment group (36 cases) according to the degree of cognitive impairment. On admission, all patients were given corresponding treatment and serum NSE detection was performed. The results showed that the severity of cognitive impairment was closely related to the level of serum NSE in patients with vascular cognitive impairment, and the more severe the degree of cognitive impairment, the higher the NSE value. Therefore, it was concluded the serum NSE level in patients with vascular cognitive impairment could be used to judge the severity of cognitive impairment (Chang, 2018).

### 2.3 Expression of NSE in AD

Patients with AD exhibit amyloid beta deposition extracellularly, leading to mitochondrial and peripheral nerve membrane damage, decreased energy production, and impaired synthesis of neurotransmitters. This cascade of events ultimately results in nerve cell dysfunction (Jahangiri et al., 2019). Following injury, neurotrophic factors are released from nerve cells into the extracellular space and can cross the blood-brain barrier into bloodstream. Consequently, measuring levels of nerve damage markers in the blood can serve as an indicator of the extent of nerve damage and disease severity in AD patients (Zhai et al., 2016; Yook and Cho, 2017; Jahangiri et al., 2019). NSE is a commonly used marker of nerve injury, and the increase of serum NSE level in AD patients is closely related to the degree of brain injury (Öhrfelt et al., 2016). At the same time, studies have shown that NSE is correlated with the classic markers of AD: amyloid beta and tau proteins (Harrington et al., 1993; Hwang et al., 2004). NSE can be considered as a further biomarker for the early diagnosis of AD (Palumbo et al., 2008).

Some researchers used electrochemical luminescence immunoassay (ECLIA) to measure the level of cerebrospinal fluid NSE in AD patients and healthy subjects, and the results showed that the level of cerebrospinal fluid NSE in AD patients was significantly higher than that in healthy subjects. It is concluded that the elevated level of CSF-NSE reflects the altered neuronal metabolism in AD, which may be used to support the diagnosis of AD (Schmidt et al., 2014). Similarly, a meta-analysis of NSE in AD showed increased levels of NSE in the cerebrospinal fluid and increased levels of NSE in the serum of patients compared to controls (Olsson et al., 2016).

### 2.4 Expression of NSE in cognitive dysfunction secondary to craniocerebral injury

During craniocerebral injury, cerebral ischemic organic damage can result in neuronal damage and neuroconduction disorders, impacting cognitive function by disrupting the connectivity between the cortex and subcortical regions. This process is influenced by various pathogenic factors, including abnormal glucose and the lipid metabolism, neuronal edema, demyelinating changes, ischemia, and hypoxia, leading to increased permeability of the blood-brain barrier and subsequent leakage of NSE (Slavoaca et al., 2020). Dana et al. investigated the use of serum NSE biomarkers to predict neurocognitive outcomes of Traumatic brain injuries (TBI) by detecting serum NSE in the first 4 h and 72 h after injury in 62 patients with moderate-to-severe TBI. Cognitive status was measured using a brief mental state examination (MMSE) at 10 and 90 days post-injury, and structural equation models (SEM) were used to assess overall neurocognitive status. 4-hour NSE values were significant predictors of cognitive status at 10 days (*p* = 0.034) and 90 days (*p* = 0.023) (Slavoaca et al., 2020). The study showed that there was a significant correlation between 4-h NSE levels and short -to medium-term neuropsychological outcomes, and that this biomarker could be used to select patients with craniocerebral injury who were at higher risk of cognitive impairment.

### 2.5 Expression of NSE in postoperative secondary cognitive dysfunction

Postoperative cognitive dysfunction (POCD) frequently occurs following general anesthesia in elderly patients, primarily due to cerebral ischemia-reperfusion injury. The underlying mechanism involves a pathophysiological cascade including excitatory amino acid release, oxidative stress, calcium overload, inflammatory response, and apoptosis. Serum levels of NSE and the glial cell marker S100β are closely associated with the extent of brain injury and subsequent recovery in this patient population (Kessler et al., 2007).

Xie and Yao (2023) conducted a study on 162 elderly patients with general anesthesia. According to whether POCD occurred 24 h after surgery, 162 elderly patients with general anesthesia were divided into POCD group and non-POCD group, and the serum NSE level was measured. At the end of surgery and 24 h after surgery, the serum NSE level in the POCD group was higher than that in the non-POCD group (all *p* < 0.001). Further analysis found that the serum NSE level in the POCD group was negatively correlated with the MMSE score 24 h after surgery, suggesting that the serum NSE level increased with the increase of the severity of cognitive dysfunction, and NSE caused cognitive dysfunction through direct or indirect mechanisms. Therefore, the level of serum NSE can effectively predict the status of POCD in elderly patients (Jones et al., 2012). Baranyi and Rothenhäusler (2013) demonstrated in their study that serum levels of NSE exhibited a sustained elevation in elderly patients with cognitive impairment following coronary artery bypass graft surgery.

### 2.6 Expression of NSE in ASD

The possible causes of cognitive dysfunction in ASD are neuroimmune abnormalities, damage of inflammatory cytokines, oxidative stress, intestinal flora disorders, and genetic changes (Liang et al., 2020; Buch et al., 2023). NSE is one of the important peripheral blood markers of neuronal injury. One study reported that 14.5% of the enolase gene (ENO2) was hypermethylated and ENO2 RNA expression was reduced by 70% in ASD patients compared to controls (Wang et al., 2014). At the same time, the children in the ENO2 hypermethylation group all had significant language expression disorders, and the researchers speculated that ENO2 had a function in language development. Multiple previous studies have reported significantly higher serum NSE levels in patients with ASD compared to healthy controls (Ayaydin et al., 2020; Stancioiu et al., 2023). Stancioiu et al. (2023) found that NSE values increased in the vast majority of children with ASD in their study, suggesting that NSE should be considered and further evaluated as an important new biomarker for ASD, which could greatly improve the diagnostic and therapeutic capabilities of this pathology.

## 3 Conclusion

**Figure 2:**
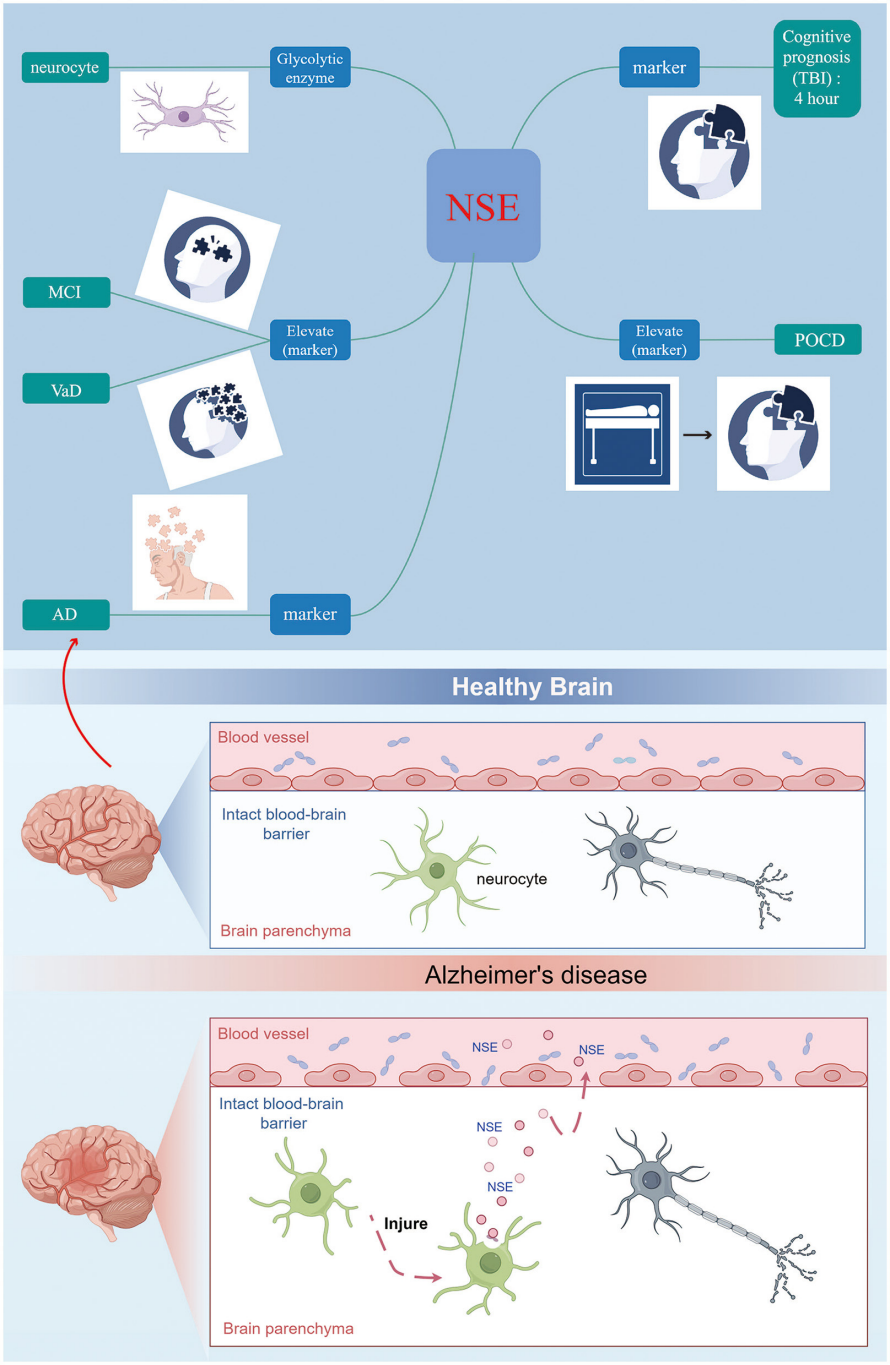
NSE can be considered as a further biomarker for the early diagnosis of cognitive disorder.

In conclusion, NSE serves as a specific biomarker indicative of neuronal damage following cerebral ischemia (Figure 2). It is ubiquitously present in the interstitial spaces of nerve and neuroendocrine cells. Upon neuronal injury, alterations in cell membrane permeability lead to the migration of NSE into the bloodstream via the blood-brain barrier, resulting in a significant elevation of NSE levels in peripheral blood (Lasek-Bal et al., 2019). Once migrating to the plasma membrane, NSE is involved in cell activation, production of inflammatory cytokines and chemokines, and induction of neuronal cell death (neurodegeneration) (Hafner et al., 2012; Haque et al., 2016). Regulated expression of NSE may promote neuronal survival (neuroprotection or regeneration) through a cell survival pathway. The utilization of NSE cell molecules as indicators of neurological impairment and their association with cognitive dysfunction is a significant area of study. The assessment of serum NSE levels, due to its simplicity, speed, and efficacy, plays a crucial role in enhancing the wellbeing of individuals with cognitive impairment. It aids in early prediction of cognitive decline, evaluation of disease severity, prognosis estimation, and implementation of targeted preventive measures. Briefly, CSF or serum NSE may be valuable biomarkers for identifying patients with cognitive impairment. Increasing the understanding of NSE and the mechanism of NSE survival and death in neuronal cells may help to find new therapeutic approaches and provide new targets for the prevention of cognitive impairment progression and future cognitive impairment.

## 4 Prospect

In the next step, the research direction of our research group is to explore the predictive value of serum NSE level in patients with cognitive dysfunction by measuring the correlation between serum NSE expression level and Montreal Cognitive Assessment Scale (MoCA) score in people at high risk of cognitive impairment, so as to judge the severity of the disease, estimate the prognosis and take targeted prevention and control measures. Improve the quality of life of patients with cognitive dysfunction.

## Author contributions

FL: Conceptualization, Funding acquisition, Investigation, Methodology, Writing – original draft. HL: Data curation, Supervision, Writing – original draft. XH: Project administration, Supervision, Validation, Writing – original draft. YL: Formal analysis, Resources, Visualization, Writing – original draft. ZY: Project administration, Software, Writing – review & editing.

## References

[B1] Angelov D. N. Neiss W. F. Gunkel A. Guntinas-Lichius O. Stennert E. (1994). Axotomy induces intranuclear immunolocalization of neuron-specific enolase in facial and hypoglossal neurons of the rat. J. Neurocytol. 23, 218–233. 10.1007/BF01275526 8035205

[B2] Ayaydin H. Kirmit A. Çelik H. Akaltun I. Koyuncu I. Bilgen Ulgar S. (2020). High serum levels of serum 100 beta protein, neuron-specific enolase, Tau, active caspase-3, M30 and M65 in children with autism spectrum disorders. Clin. Psychopharmacol. Neurosci. 18, 270–278. 10.9758/cpn.2020.18.2.270 32329316 PMC7242104

[B3] Baranyi A. Rothenhäusler H. B. (2013). The impact of S100b and persistent high levels of neuron-specific enolase on cognitive performance in elderly patients after cardiopulmonary bypass. Brain Inj. 27, 417–424. 10.3109/02699052.2012.750751 23473361

[B4] Bock E. Dissing J. (2010). Demonstration of enolase activity connected to the brain-specific protein 14-3-2. Scand. J. Immunol. 4, 31–36. 10.1111/j.1365-3083.1975.tb03806.x

[B5] Buch A. M. Vértes P. E. Seidlitz J. Kim S. H. Grosenick L. Liston C.. (2023). Molecular and network-level mechanisms explaining individual differences in autism spectrum disorder. Nat. Neurosci. 26, 650–663. 10.1038/s41593-023-01259-x 36894656 PMC11446249

[B6] Butterfield D. A. Lange M. L. (2009). Multifunctional roles of enolase in Alzheimer's disease brain: beyond altered glucose metabolism. J. Neurochem. 111, 915–933. 10.1111/j.1471-4159.2009.06397.x 19780894 PMC4454338

[B7] Chang Q. L. (2018). Changes of serum levels of NSE, GFAP and BDNF in patients with vascular cognitive impairment. Labeled Immunoassays Clin. Med. 25, 517–520. 10.11748/bjmy.issn.1006-1703.2018.04.020

[B8] Díaz-Ramos A. Roig-Borrellas A. García-Melero A. López-Alemany R. (2012). α-Enolase, a multifunctional protein: its role on pathophysiological situations. J. Biomed. Biotechnol. 2012:156795. 10.1155/2012/156795 23118496 PMC3479624

[B9] Dobrynina L. A. Tsypushtanova M. M. Shabalina A. A. (2023). Biochemical markers of neurodegeneration in patients with cerebral small vessel disease and Alzheimer's disease. Ann. Clin. Exp. Neurol. 17, 21–30. 10.54101/ACEN.2023.3.3

[B10] González-Quevedo A. García S. G. Concepción Q. F. Freixas R. S. Sotolongo L. Q. Menéndez M. C.. (2011). Increased serum S-100B and neuron specific enolase - potential markers of early nervous system involvement in essential hypertension. Clin. Biochem. 44, 154–159. 10.1016/j.clinbiochem.2010.11.006 21130083

[B11] Hafner A. Obermajer N. Kos J. (2012). γ-Enolase C-terminal peptide promotes cell survival and neurite outgrowth by activation of the PI3K/Akt and MAPK/ERK signalling pathways. Biochem. J. 443, 439–450. 10.1042/BJ20111351 22257123

[B12] Haque A. Ray S. K. Cox A. Banik N. L. (2016). Neuron specific enolase: a promising therapeutic target in acute spinal cord injury. Metab. Brain Dis. 31, 487–495. 10.1007/s11011-016-9801-6 26847611 PMC4864119

[B13] Harrington C. R. Quinn G. B. Hurt J. Wischik C. M. (1993). Characterisation of an epitope specific to the neuron-specific isoform of human enolase recognised by a monoclonal antibody raised against a synthetic peptide corresponding to the C-terminus of beta/A4-protein. Biochim. Biophys. Acta. 1158, 120–128. 10.1016/0304-4165(93)90005-S 7691181

[B14] Hein-Née M. K. Kohler A. Diem R. Sättler M. B. Demmer I. Lange P.. (2008). Biological markers for axonal degeneration in CSF and blood of patients with the first event indicative for multiple sclerosis. Neurosci. Lett. 436, 72–76. 10.1016/j.neulet.2008.02.064 18359164

[B15] Hu H. Y. Ou Y. N. Shen X. N. Qu Y. Ma Y. H. Wang Z. T.. (2021). White matter hyperintensities and risks of cognitive impairment and dementia: a systematic review and meta-analysis of 36 prospective studies. Neurosci. Biobehav. Rev. 120, 16–27. 10.1016/j.neubiorev.2020.11.007 33188821

[B16] Hwang D. Y. Cho J. S. Lee S. H. Chae K. R. Lim H. J. Min S. H.. (2004). Aberrant expressions of pathogenic phenotype in Alzheimer's diseased transgenic mice carrying NSE-controlled APPsw. Exp. Neurol. 186, 20–32. 10.1016/j.expneurol.2003.09.021 14980807

[B17] Isgrò M. A. Bottoni P. Scatena R. (2015). Neuron-specific enolase as a biomarker: biochemical and clinical aspects. Adv. Exp. Med. Biol. 867, 125–143. 10.1007/978-94-017-7215-0_9 26530364

[B18] Jahangiri Z. Gholamnezhad Z. Hosseini M. (2019). Neuroprotective effects of exercise in rodent models of memory deficit and Alzheimer's. Metab. Brain Dis. 34, 21–37. 10.1007/s11011-018-0343-y 30443769

[B19] Jones E. L. Gauge N. Nilsen Q. B. Lowery D. Wesnes K. Katsaiti E.. (2012). Analysis of neuron-specific enolase and S100B as biomarkers of cognitive decline following surgery in older people. Dement. Geriatr. Cogn. Disord. 34, 307–311. 10.1159/000345538 23208248

[B20] Juva K. Sulkava R. Erkinjuntti T. Ylikoski R. Valvanne L. Tilvis R.. (1994). Staging the severity of dementia: comparison of clinical (CDR, DSM-III-R), functional (ADL, IADL) and cognitive (MMSE) scales. Acta Neurol. Scand. 90, 293–298. 10.1111/j.1600-0404.1994.tb02724.x 7839817

[B21] Kessler F. H. Woody G. Portela L. V. Tort A. B. Boni R. D. Peuker A. C.. (2007). Brain injury markers (S100B and NSE) in chronic cocaine dependents. Braz. J. Psychiatry 29, 134–139. 10.1590/S1516-44462006005000029 17650535

[B22] Lasek-Bal A. Jedrzejowska-Szypulka H. Student S. Warsz-Wianecka A. Zareba K. Puz P.. (2019). The importance of selected markers of inflammation and blood-brain barrier damage for short-term ischemic stroke prognosis. J. Physiol. Pharmacol. 70, 209–217. 10.1101/503953 31356182

[B23] Lecordier S. Manrique-Castano D. El Moghrabi Y. ElAli A. (2021). Neurovascular alterations in vascular dementia: emphasis on risk factors. Front. Aging Neurosci. 13:727590. 10.3389/fnagi.2021.727590 34566627 PMC8461067

[B24] Liang Y. Xiao Z. Ke X. Yao P. Chen Y. Lin L.. (2020). Urinary metabonomic profiling discriminates between children with autism and their healthy siblings. Med. Sci. Monit. 26:e926634. 10.12659/MSM.926634 33237888 PMC7702663

[B25] McCoy H. M. Polcyn R. Banik N. L. Haque A. (2023). Regulation of enolase activation to promote neural protection and regeneration in spinal cord injury. Neural. Regen. Res. 18, 1457–1462. 10.4103/1673-5374.361539 36571342 PMC10075133

[B26] Morley J. E. (2018). An overview of cognitive impairment. Clin. Geriatr. Med. 34, 505–513. 10.1016/j.cger.2018.06.003 30336985

[B27] Öhrfelt A. Johansson P. Wallin A. Andreasson U. Zetterberg H. Blennow K.. (2016). Increased cerebrospinal fluid levels of ubiquitin carboxyl-terminal hydrolase L1 in patients with Alzheimer's disease. Dement. Geriatr. Cogn. Dis. Extra. 6, 283–294. 10.1159/000447239 27504117 PMC4965532

[B28] Olsson B. Lautner R. Andreasson U. Öhrfelt A. Portelius E. Bjerke M.. (2016). CSF and blood biomarkers for the diagnosis of Alzheimer's disease: a systematic review and meta-analysis. Lancet Neurol. 15, 673–684. 10.1016/S1474-4422(16)00070-3 27068280

[B29] Palumbo B. Siepi D. Sabalich I. Tranfaglia C. Parnetti L. (2008). Cerebrospinal fluid neuron- specific enolase: a further marker of Alzheimer's disease? Funct. Neurol. 23, 93–96. 18671910

[B30] Petersen R. C. (2004). Mild cognitive impairment as a diagnostic entity. J. Intern. Med. 256, 183–194. 10.1111/j.1365-2796.2004.01388.x 15324362

[B31] Polyakova M. Mueller K. Arelin K. Lampe L. Rodriguez F. S. Luck T.. (2022). Increased serum NSE and S100B indicate neuronal and glial alterations in subjects under 71 years with mild neurocognitive disorder/mild cognitive impairment. Front. Cell Neurosci. 16:788150. 10.3389/fncel.2022.788150 35910248 PMC9329528

[B32] Rajeev V. Fann D. Y. Dinh Q. N. Kim H. A. Silva M. D. Lai M. K.. (2022). Pathophysiology of blood brain barrier dysfunction during chronic cerebral hypoperfusion in vascular cognitive impairment. Theranostics 12, 1639–1658. 10.7150/thno.68304 35198062 PMC8825579

[B33] Sacrey L. A. Germani T. Bryson S. E. Zwaigenbaum L. (2014). Reaching and grasping in autism spectrum disorder: a review of recent literature. Front. Neurol. 5:6. 10.3389/fneur.2014.00006 24478753 PMC3899541

[B34] Schmechel D. E. Brightman M. W. Marangos P. J. (1980). Neurons switch from non-neuronal enolase to neuron-specific enolase during differentiation. Brain Res. 190, 195–214. 10.1016/0006-8993(80)91169-5 6769533

[B35] Schmidt F. M. Mergl R. Stach B. Jahn I. Gertz H. J. Schönknecht P. (2014). Elevated levels of cerebrospinal fluid neuron-specific enolase (NSE) in Alzheimer's disease. Neurosci. Lett. 570, 81–85. 10.1016/j.neulet.2014.04.007 24746933

[B36] Shen Y. Gao H. M. (2015). Serum somatostatin and neuron-specific enolase might be biochemical markers of vascular dementia in the early stage. Int. J. Clin. Exp. Med. 8, 19471–19475. 26770594 PMC4694494

[B37] Slavoaca D. Birle C. Stan A. Tatomir A. Popa O. Rosu P.. (2020). Prediction of neurocognitive outcome after moderate-severe traumatic brain injury using serum neuron-specific enolase and s100 biomarkers. J. Med. Life 13, 306–313. 10.25122/jml-2020-0147 33072201 PMC7550145

[B38] Stancioiu F. Bogdan R. Dumitrescu R. (2023). Neuron-specific enolase (NSE) as a biomarker for autistic spectrum disease (ASD). Life 13:1736. 10.3390/life13081736 37629593 PMC10455327

[B39] Tanaka M. Sugisaki K. Nakashima K. (1985). Switching in levels of translatable mRNAs for enolase isozymes during development of chicken skeletal muscle. Biochem. Biophys. Res. Commun. 133, 868–72. 10.1016/0006-291X(85)91215-X 4084308

[B40] Tian R. Jiang Y. Zhang Y. Yan X. Zhou Y. Chen D.. (2022). Cognitive training program improves cognitive ability and daily living ability in elderly patients with mild cognitive impairment. Aging Clin. Exp. Res. 34, 997–1005. 10.1007/s40520-021-02015-6 34767246

[B41] Wallin A. Blennow K. Rosengren L. (1999). Cerebrospinal fluid markers of pathogenetic processes in vascular dementia, with special reference to the subcortical subtype. Alzheimer Dis. Assoc. Disord. 13, S102–S105. 10.1097/00002093-199912001-00015 10609688

[B42] Wang Y. Fang Y. Zhang F. Xu M. Zhang J. Yan J.. (2014). Hypermethylation of the enolase gene (ENO2) in autism. Eur. J. Pediatr. 173, 1233–44. 10.1007/s00431-014-2311-9 24737292 PMC4134484

[B43] Xie Y. Yao Z. (2023). Relationships of serum VILIP-1, NSE, and ADP levels with postoperative cognitive dysfunction in elderly patients undergoing general anesthesia:a retrospective, observational study. J. Int. Med. Res. 51:3000605231172447. 10.1177/03000605231172447 37194201 PMC10192666

[B44] Yook J. S. Cho J. Y. (2017). Treadmill exercise ameliorates the regulation of energy metabolism in skeletal muscle of NSE/PS2mtransgenic mice with Alzheimer's disease. J. Exerc. Nutr. Biochem. 21, 40–47. 10.20463/jenb.2017.0046 28712264 PMC5508058

[B45] Zhai Y. Yamashita T. Nakano Y. Sun Z. Morihara R. Fukui Y.. (2016). Disruption of white matter integrity by chronic cerebral hypoperfusion in Alzheimer's disease mouse model. J. Alzheimers Dis. 52, 1311–1319. 10.3233/JAD-160120 27079724

